# Metagenomic Insights into Microbial Signatures in Thrombi from Acute Ischemic Stroke Patients Undergoing Endovascular Treatment

**DOI:** 10.3390/brainsci15020157

**Published:** 2025-02-06

**Authors:** Kasthuri Thirupathi, Sherief Ghozy, Abdullah Reda, Wasantha K. Ranatunga, Mars A. Ruben, Zarrintan Armin, Oana M. Mereuta, Sekhon Prabhjot, Daying Dai, Waleed Brinjikji, David F. Kallmes, Ramanathan Kadirvel

**Affiliations:** 1 Department of Neurological Surgery, Mayo Clinic, Rochester, MN 55905, USA; thirupathi.kasthuri@mayo.edu (K.T.); mohammed.abdullah@mayo.edu (A.R.); ranatunga.wasantha@mayo.edu (W.K.R.); mereuta.oana@mayo.edu (O.M.M.); 2 Department of Radiology, Mayo Clinic, Rochester, MN 55905, USA; dai.daying@mayo.edu (D.D.); brinjikji.waleed@mayo.edu (W.B.); kallmes.david@mayo.edu (D.F.K.); 3 Division of Gastroenterology and Hepatology, Department of Medicine, Mayo Clinic, Rochester, MN 55905, USAsekhon.prabhjotkaur@mayo.edu (S.P.)

**Keywords:** acute ischemic stroke, thrombus microbiota, mechanical thrombectomy, 16S ribosomal RNA sequencing, bacterial infections

## Abstract

**Background:** Variability in recanalization success during endovascular treatment for acute ischemic stroke (AIS) has led to increased interests in thrombus composition and associated cellular materials. While evidence suggests that bacteria may influence thrombus characteristics, limited data exist on microbiological profiles of thrombi in stroke patients. **Objectives:** Characterization of bacterial communities present in thrombi of AIS patients undergoing mechanical thrombectomy, providing insights into microbial contributions to stroke pathogenesis and treatment outcomes. **Methods:** Thrombi were collected from 20 AIS patients. After extracting metagenome, 16S rDNA sequencing was performed. Bioinformatic analysis included taxonomy and diversity assessments. The presence of bacterial DNA and viable bacteria in thrombi was validated using polymerase chain reaction (PCR) and bacterial culturing followed by matrix-assisted laser desorption ionization–time of flight (MALDI-TOF) analysis, respectively. **Results:** 16S rDNA was amplified in 19/20 thrombi (95%). Analysis identified a diverse microbial community, with *Corynebacterium* spp. as the most prevalent genus, followed by *Staphylococcus* spp., *Bifidobacterium* spp., *Methylobacterium* spp., and *Anaerococcus* spp. Alpha diversity analyses (Shannon index: 4.0–6.0 and Simpson index: 0.8–1.0) revealed moderate to high microbial diversity across samples; beta diversity demonstrated distinct clustering, indicating inter-patient variability in microbial profiles. PCR confirmed the presence of DNA specific to dominant bacterial taxa identified through sequencing. Culturing showed the presence of *Staphylococcus epidermidis* and *Enterococcus faecalis* in some clots as identified through MALDI analysis. **Conclusions:** This study shows bacterial communities present in AIS patients’ thrombi, suggesting a potential link between microbial signatures and thrombus characteristics.

## 1. Introduction

Successful recanalization in acute ischemic stroke (AIS) caused by large vessel occlusion (LVO) varies significantly depending on patient-specific factors and thrombus characteristics. Thrombi composition has been a recent area of research to better understand how the application of different endovascular treatments can impact patient outcomes in relation to thrombus composition. Among various factors proposed to affect clot characteristics, bacterial inflammation has been suggested as one of the key factors due to evidence implying its role in the development of atherothrombotic and atherosclerotic events [1]. In addition, there have been multiple studies that documented the interaction between gut microbiota and stroke pathogenesis [2,3,4,5,6].

Endovascular treatment offers a unique opportunity to investigate thrombi retrieved from patients with LVO stroke, including the thrombus-associated microbiota [7,8,9]. Recent studies have shown that the presence of microbiota in myocardial infarction patients’ thrombi may influence the course of thrombosis and the likelihood of severe adverse cardiovascular events [10,11,12,13]. However, prior to endovascular mechanical thrombectomy becoming recognized as the first-line treatment for AIS with LVO, there was a paucity of information regarding the microbiological analysis of thrombi from stroke patients [14,15,16,17,18,19]. Although some recent studies reported the presence of bacteria in AIS patients’ clots [9,20,21], the sample size of these studies underscores the need to conduct further investigations to understand the prevalence of these organisms and potentially identify additional species that might have an impact on thrombus composition and patient outcomes. This study was conducted to investigate the presence of bacterial species in thrombi retrieved after thrombectomy for patients with AIS.

## 2. Materials and Methods

### 2.1. Subjects and Specimen Collection

Institutional review board approval was obtained, and a waiver of consent was granted. The inclusion criteria were patients aged 18 years or older who had received mechanical thrombectomy for acute ischemic stroke, with clot material successfully retrieved during the procedure.

Samples were obtained from 20 patients without gender-based selection. Following the procedure, the collected thrombus specimens were placed in sterile saline and promptly transported to the research laboratory, where they were stored at −80 °C until further processing. For the purpose of this study, we gathered data on patient demographics, comorbidities, the administration of tissue plasminogen activator (tPA), the location of the occluded vessel, the number of thrombectomy passes, and the final Thrombolysis in Cerebral Infarction (TICI) score.

### 2.2. Metagenomic DNA Extraction, Illumina Sequencing, and Bioinformatic Analysis

DNA from patient clots was extracted using the DNeasy Blood & Tissue Kit (QIAGEN, Germantown, MD, USA), following the manufacturer’s protocol. The quality and quantity of the extracted DNA were verified using a NanoDrop spectrophotometer (Thermo Fisher Scientific, Waltham, MA, USA) and agarose gel electrophoresis. To control the potential contamination during DNA isolation, phosphate-buffered saline was used as a negative control. The negative control was processed and analyzed alongside the patient samples to monitor the potential contamination throughout the extraction and sequencing processes. The presence of the 16S rDNA region was verified via polymerase chain reaction (PCR) using primers 27F and 1392R. Sequencing of the 16S rDNA V4 region amplicons was performed at the University of Minnesota Genomics Center using the Illumina MiSeq platform, with 2 × 300 bp paired-end sequencing chemistry.

Subsequent bioinformatic analysis including the removal of primer sequences using Atropos, followed by trimming and denoising with DADA2. Phylogenetic trees were constructed using FastTree (version Version: 2.1.11). Taxonomic assignment was conducted using the SILVA v132 16S sequence database. Further filtering was performed using SortMeRNA (version 4.3.7) and Infernal (version 1.1.5).

### 2.3. Biostatistical Analysis

Taxa tables generated from the sequencing data were analyzed using R (version 4.4.0, R Foundation for Statistical Computing, Vienna, Austria). A phyloseq object was created to ensure consistent sample naming and data integrity, with pseudocounts added to prevent zero values. Character variables were converted to factors that were required by DESeq2 for appropriate statistical handling.

Prior to formal analysis, we performed a double layer of quality control to avoid false positive results. First, we excluded any taxa (ASVs) that were identified in the control sample and thus potentially originated from handling and storing conditions of the patient samples. Second, we excluded any identified ASVs below the 25th percentile of total bacterial counts across samples. Following this filtering process, rarefaction analysis was conducted using the rarefy_even_depth function in the phyloseq package to evaluate sampling depth and taxonomic richness. Alpha diversity metrics, including Shannon and Simpson indices, were calculated and visualized using bar plots. Significance was determined at a *p*-value threshold of 0.05 for all statistical analyses. Beta diversity was assessed through Principal Coordinates Analysis (PCoA) based on Bray–Curtis dissimilarity distances, and the results were visualized with sample labels to clarify the position of each sample within the diversity space.

### 2.4. Validation of Presence of Species-Specific Genes

Qualitative PCR was performed to confirm the presence of species-specific bacterial genes identified in 16S rDNA sequencing. Specific primers targeting these species along with a housekeeping gene (GAPDH) are detailed in Table S1.

### 2.5. In Vitro Bacterial Culture

To assess the presence of viable bacteria in stroke patient clot samples, in vitro bacterial culturing was performed in the Brain Heart Infusion (BHI) broth in different patient clots (n = 10). Samples were added to the 2 mL of BHI broth and incubated at 37 °C for 72 h with 160 rpm. After incubation, 100 µL was spread on the 5% sheep blood agar and then incubated at 37 °C for 24 h. Individual colonies were picked, sub-cultured, and subjected to species identification.

### 2.6. Species Identification Using MALDI

Individual colonies from 5% sheep blood agar were picked and spotted four times onto a MALDI target plate and then allowed to air dry at room temperature. Each sample spot was subsequently overlaid with 1 μL of a Bruker matrix solution HCCA (α-cyano-4-hydroxycinnamic acid in a mixture of acetonitrile, water, and trifluoroacetic acid). The target plate also included a control spot containing pure matrix solution as negative control and another spot containing the Bacterial Test Standard (Bruker Daltonics GmbH & Co. KG, Bremen, Germany) for calibration purposes. The analysis was performed automatically using a MALDI Bruker Biotyper Sirus system (Bruker Daltonics GmbH & Co. KG). Spectra were collected with FlexControl (v3.4) software, adhering to the manufacturer’s guidelines (Bruker Daltonics GmbH & Co. KG, Germany). Species identification was conducted by comparing the obtained mass spectra against reference spectra from the BDAL database (Bruker Daltonics GmbH & Co. KG, Germany). For this dataset, species labels were assigned based on the highest match found between the generated mass spectra and those in the BDAL database. Mass spectra that did not achieve a Bruker log score above 1.8 were excluded from the final dataset.

### 2.7. Fluorescence In Situ Hybridization (FISH) Analysis

Fluorescence in situ hybridization (FISH) analysis was performed on patient clot tissues and clot analogs to evaluate bacterial presence and localization. Tissue samples were fixed in 10% neutral-buffered formalin, processed using an automated tissue processor (Leica ASP 300S, Leica, Wetzlar, Germany), and embedded in paraffin blocks. Thin sections (3–4 μm) were prepared for FISH staining. The staining procedure was carried out on a Bond Max auto-stainer (Leica) and included several steps: deparaffinization using Bond Dewax Solution (Leica AR922), antigen retrieval with an EDTA-based buffer (Bond Epitope Retrieval Solution 2) heated for 20 min, and enzymatic digestion with Proteinase K (Leica AR9551) at 37 °C for 20 min to enhance probe penetration. Tissue sections were then denatured at 95 °C for 10 min, followed by hybridization with a Cy3-labeled Eubacteria-specific FISH probe (MBD0033, 2 μg/mL) at 46 °C for 2 h. Post-hybridization washes were conducted using Leica Wash Buffer (DS9347) to remove excess probes, and nuclei were counterstained with Hoechst 33258 (Invitrogen, Waltham, MA, USA). Positive controls were prepared by incorporating cultured bacteria into clot analogs, while negative controls included clot ana-logs without bacteria to confirm the absence of nonspecific hybridization. Fluorescence images were captured using an Olympus confocal fluorescence microscope with appropriate filters for Cy3 and Hoechst detection.

## 3. Results

### 3.1. Patient Characteristics

The study included 20 patients, comprising 10 females and 10 males. The majority of the patients were White (n = 18), with one Black or African American patient and one patient identified as ‘Other’. All but one patient identified as not Hispanic or Latino, with the exception being a Central American female. The age of the cohort was 70 years in median (Q1:61, Q3:77), with ages ranging from 38 to 91 years (Table 1).

Smoking status was documented for all patients, with 15 patients (75%) being current smokers. Regarding comorbidities, hypertension was prevalent in 75% of the patients (n = 15), while diabetes mellitus and hyperlipidemia were observed in two (10%) and eleven (55%) patients, respectively. Coronary artery disease was documented in six patients (30%), atrial fibrillation in ten (50%), and congestive heart failure in five patients (25%). Chronic obstructive pulmonary disease (COPD) was present in five patients (25%), and three patients (15%) had a history of previous stroke.

The occlusion sites included the middle cerebral artery (M1) in eight patients (40%), internal carotid artery (ICA) in two patients (10%), M2 segment of the middle cerebral artery in four patients (20%), and the basilar artery in one patient (5%). Treatment with tissue plasminogen activator (tPA) was administered to eight patients (40%) as part of their management plan. The number of thrombectomy passes varied across patients, with most requiring a single pass (n = 11, 55%), while others needed up to six passes. The degree of recanalization showed successful outcomes with TICI-3 in 75% patients and TICI-2B in 5% of the patients.

Notably, the majority of patients (80%) presented with thromboembolism as the primary stroke etiology, while a smaller proportion had atherosclerosis (15%) or combined atherosclerosis and distal emboli (5%). Biomarkers of inflammation, particularly leukocytosis, were evident in 40% of patients, while markers such as increased CRP and lactate were less commonly observed. A history of antibiotic use during admission was noted in 20% of the cohort, with cefepime, vancomycin, and cefazolin among the antibiotics administered. Immunodeficiency was identified in select cases, with breast cancer being the most frequent condition (10%), while positive blood cultures revealed Candida and Staphylococcus epidermidis infections in isolated cases (Table 2).

### 3.2. Microbial Composition of Thrombi

A total of 20 thrombi from AIS patients, collected through mechanical thrombectomy, were analyzed using next-generation sequencing to identify bacterial signatures. The 16S rDNA region was successfully amplified in 19 out of 20 (95%) cases, with one subject showing no amplification. No amplicons were observed in negative controls, indicating no contamination (Supplementary Figure S1). The sequencing depth varied across samples, with read counts ranging from 97 to 5890. A control sample was used to identify and filter out potential background contaminants (Table S2).

### 3.3. Taxonomic Composition and Species Diversity

**Figure 1:**
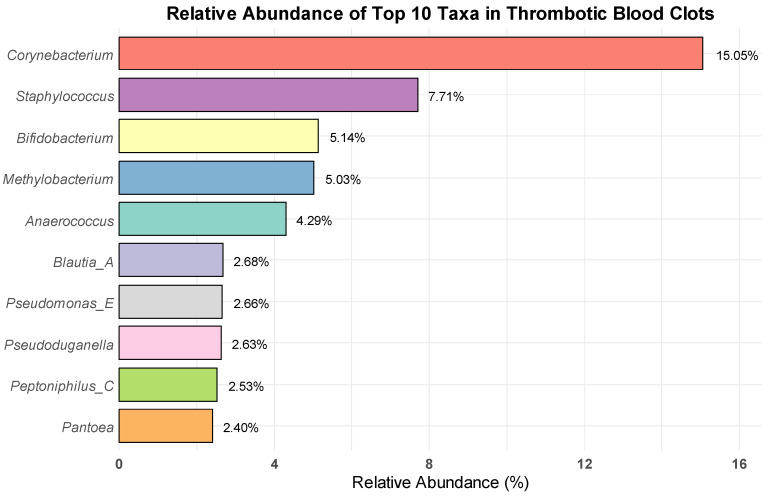
Relative abundance of the 10 most prevalent bacterial taxa in thrombotic blood clots.

Following the filtering of the data, bacterial taxa meeting our criteria were identified in 17 of the 20 examined samples. Taxa tables before and after filtering are given in the Supplementary Table S3. The analysis of bacterial taxa in thrombotic blood clots identified *Corynebacterium* spp. as the most predominant genus across all samples, with a taxonomic abundance of 1884 counts, comprising approximately 15.05% of the total microbial community. *Staphylococcus* was the second most prevalent genus, with 964 counts, representing 7.71% of the microbial composition. Other notable genera included *Bifidobacterium* (644 counts; 5.14%), *Methylobacterium* (629 counts; 5.03%), *Anaerococcus* (537 counts; 4.29%), *Blautia_A* (335 counts; 2.68%), *Pseudomonas_E* (333 counts; 2.66%), *Pseudoduganella* (330 counts; 2.63%), *Peptoniphilus C* (316 counts; 2.53%), and *Pantoea* (300 counts; 2.40%). Collectively, these 10 genera accounted for a substantial portion of the total bacterial community across all samples, amounting to 50.12% of the total (Figure 1). Detailed proportions of bacterial taxa within each sample are provided in Table S4.

### 3.4. Alpha Diversity

**Figure 2:**
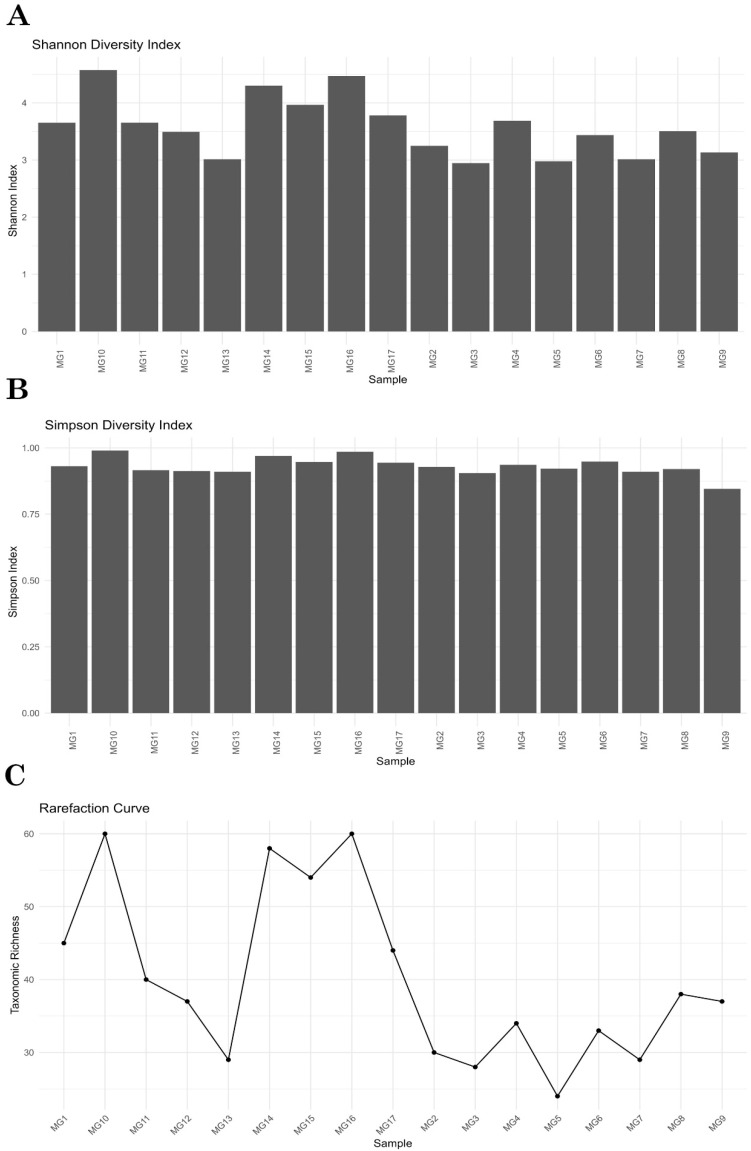
Alpha diversity indices for each sample. (**A**) Shannon diversity index; (**B**) Simpson diversity index; (**C**) Rarefaction curves showing taxonomic richness across all samples.

To evaluate the diversity within each sample, the Shannon and Simpson diversity indices were calculated. The Shannon index values ranged from 4.0 to 6.0, reflecting moderate to high diversity across the samples (Figure 2A). Three cases exhibited the highest diversity, whereas two cases showed relatively lower diversity. The Simpson index values, which account for both richness and evenness, were close to 1.0 for most samples, suggesting a highly diverse and evenly distributed microbial community (Figure 2B). Detailed alpha diversity indices for each sample are provided in Table S5.

A rarefaction analysis was performed to evaluate the adequacy of the sequencing depth in capturing microbial diversity. The rarefaction curves demonstrated varying levels of richness across the samples, with three cases showing the highest richness, while two cases exhibited the lowest (Figure 2C). This analysis confirms that the sequencing depth was sufficient for most samples, although some variation in richness was observed.

### 3.5. Beta Diversity

**Figure 3:**
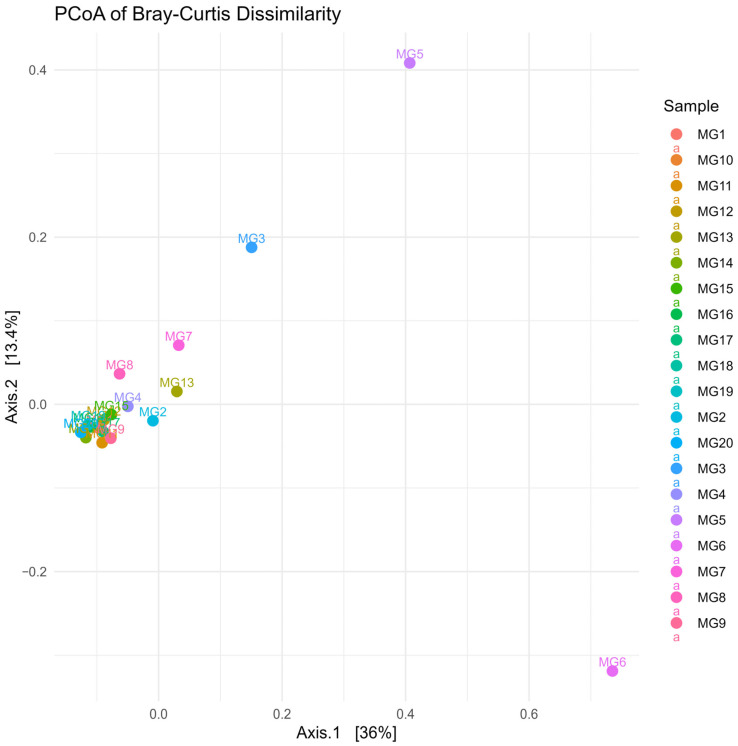
Principal Coordinates Analysis (PCoA) plot based on Bray–Curtis dissimilarity distances.

Beta diversity was assessed using Principal Coordinates Analysis (PCoA) based on Bray–Curtis dissimilarity distances. The PCoA plot revealed distinct clustering patterns among the samples, indicating differences in microbial community composition between individual samples (Figure 3). Notably, two subjects showed significant separation from the rest, suggesting unique microbial profiles that may correlate with specific clinical conditions or patient characteristics. Beta diversity distances among samples are detailed in Table S6.

### 3.6. Species Confirmation

PCR was conducted to confirm the presence of the most abundant bacterial species identified in the metagenomic analysis. The results of the PCR further validated the taxonomic findings, supporting the significant presence of *Corynebacterium* spp. and other prevalent genera.

### 3.7. In Vitro Culturing of Bacteria

Bacterial culturing assesses the presence of viable bacteria in thrombi samples. Bacterial growth was observed in two samples among the cultured ten samples. Individual colonies were subjected to MALDI-TOF analysis. Mass spectra comparison against the BDAL database revealed that the bacteria cultured from two samples are *Staphylococcus epidermidis* and *Enterococcus faecalis* with the match score of 1.89 to 1.98.

### 3.8. Fluorescence In Situ Hybridization (FISH)

The FISH analysis confirmed the presence of bacterial sequences within patient clot tissues, as evidenced by hybridization signals detected in the samples (Figure S2C). The functionality of the Cy3-labeled Eubacteria-specific probe was validated through positive hybridization signals observed in the bacterial clot analog (Figure S2A). Specificity was further confirmed by the absence of hybridization signals in the negative control clot analog, demonstrating a lack of nonspecific binding (Figure S2B).

## 4. Discussion

This study characterizes the bacterial communities present in thrombi retrieved from patients with AIS undergoing mechanical thrombectomy. The successful amplification of the 16S rDNA region in 95% of the subjects, coupled with the absence of amplicons in negative controls, suggests that there is bacterial DNA in these clots. Further, culturing results show that for at least some clots there are low numbers of live bacteria present. This study is among the first to extensively characterize the bacterial communities within stroke-related thrombi using 16S rDNA sequencing, providing new insights into the potential microbial contributions to thrombus formation and stroke pathogenesis. Our results demonstrate a diverse bacterial presence in these thrombi, with *Corynebacterium* spp. emerging as the most prevalent genus, followed by *Staphylococcus*, *Bifidobacterium*, *Methylobacterium,* and *Anaerococcus.* Other prevalent bacteria include *Pseudomonas_E*, *Pseudoduganella*, *Peptoniphilus_C*, and *Pantoea.*

Previous research has highlighted the potential role of bacterial pathogens in the development of atherosclerosis and other thrombotic events [22,23]. The presence of bacterial DNA within thrombi, as identified in this study, suggests a potential link between microbial activity and atherosclerotic plaque rupture. Infectious processes have long been hypothesized to contribute to the initiation and progression of atherosclerosis, particularly through chronic low-grade inflammation and direct endothelial injury [24]. Bacterial components, such as lipopolysaccharides (LPS) from Gram-negative bacteria, are known to activate Toll-like receptors (TLRs) on endothelial cells and macrophages, triggering inflammatory signaling cascades that lead to increased production of pro-inflammatory cytokines, including tumor necrosis factor-alpha (TNF-α), interleukin-6 (IL-6), and interleukin-1 beta (IL-1β) [25,26,27]. These cytokines, in turn, contribute to endothelial dysfunction, oxidative stress, and smooth muscle cell proliferation—hallmarks of atherosclerotic plaque progression [25,26,28,29]. Furthermore, bacterial colonization within plaques has been implicated in enhancing plaque instability. Previous studies have detected DNA from various bacterial species within atherosclerotic lesions, suggesting that bacterial infiltration may be more than an incidental finding [30,31,32].

Our findings provide additional evidence supporting the role of bacterial presence in thrombus composition and stroke pathogenesis. The identification of *Corynebacterium* spp. in our study aligns with existing literature suggesting an association between this genus and infective endocarditis, particularly in immunocompromised individuals or those with significant comorbidities [33,34]. Despite its usual classification as non-pathogenic or a contaminant, *Corynebacterium* spp. has been implicated in serious infections, including infective endocarditis, especially in older, hospitalized patients with underlying conditions [35,36,37,38,39]. This demographic profile matches the characteristics of our patient cohort, potentially explaining the prominence of this genus in our samples. The demographic profile of patients with infective endocarditis, particularly those with prosthetic valves or who have undergone invasive procedures [33,40], matches the characteristics of our patient cohort, potentially explaining the prominence of this genus in our samples.

Our findings also corroborate earlier studies documenting bacterial DNA in thrombi from stroke patients. For instance, the frequent detection of *Staphylococcus* spp. in our samples is noteworthy, particularly given their known role in thrombus formation and immune evasion. Recent findings have demonstrated that coagulase-positive *Staphylococcus aureus* can induce clotting of plasma, serving as a mechanism to persist within the fibrin network and evade host immune responses [41]. This ability to promote clotting and stabilize within a thrombus could contribute to the formation and resilience of the clots observed in AIS patients.

It is noteworthy that some previously detected bacteria did not appear among the dominant taxa in our analysis. For instance, *Streptococcus* spp. was found in a significant percentage of thrombi in previous studies, though it did not appear among the dominant taxa in our analysis [9,20,42]. The diversity of bacterial communities, as indicated by our alpha and beta diversity analyses, reflects the heterogeneity observed in other investigations, suggesting that the microbial composition of thrombi vary significantly among individuals. This variability might be influenced by factors such as patient comorbidities, the site of occlusion, and the number of thrombectomy passes. It is also important to note that some previous literature has found no evidence of bacterial DNA in thrombi retrieved from AIS patients [8]. A key difference between that work and ours lies in the method of clot preservation. The previous research used formalin fixation, which, while preserving tissue structure, may have degraded bacterial DNA, potentially leading to false-negative results [8]. In contrast, our study utilized frozen clots, a method that better preserves nucleic acids, thus enhancing the detection of microbial communities. This methodological difference likely accounts for the discrepancy in findings and underscores the importance of preservation techniques in microbial detection using metagenome sequencing.

The implications of bacterial presence in thrombi on stroke outcomes remain an area for further research. Understanding whether these bacteria contribute to clot stability or influence the success of mechanical thrombectomy could inform treatment strategies and improve patient prognosis. For example, the interaction between bacterial components and platelet receptors, as suggested by our findings, could potentially affect thrombus formation, making clots more resistant to conventional treatments [43,44]. Among the most prevalent bacteria identified in our samples, *Pseudomonas_E*, *Pseudoduganella,* and *Pantoea* are Gram-negative genera. It is well-established that bacterial components, such as lipopolysaccharides from Gram-negative bacteria, can interact with platelet receptors, leading to platelet activation and aggregation [45]. Moreover, bacterial disulfide bond proteins, homologous to human protein disulfide isomerase, may contribute to coagulation processes and thrombus stability [46,47,48]. These interactions suggest a potential mechanism by which bacterial presence within thrombi could influence stroke severity and recovery. In addition, the present study identified the presence of *S. epidermidis* and *E. faecalis* in two different clot samples. During the stationary phase growth cycle, the *S. epidermidis* cell surface adhesion protein SdrG binds to host fibrinogen, resulting in the formation of a fibrin-rich complex network which increases the thrombus elasticity (28). Human intracellular signal transduction pathways key regulator, human protein four, and a half LIM domains protein 2 (FHL2) deficiency enhances the human venous thrombus formation (29). Virulence factor ElrA of *E. faecalis* directly targets the FHL2 (30). From this study, it is a speculation that binding of ElrA could compete with the availability of FHL2 for signal transduction pathways, resulting in the venous thrombus formation.

However, several limitations should be acknowledged. The relatively small sample size may limit the generalizability of our findings, and the observed microbial diversity might exaggerate the prevalence of certain taxa. Beyond the sample size, another limitation to consider is the potential for contamination during sample collection and processing. While we took measures to ensure the accuracy of our results, including negative controls, the possibility of environmental or procedural contamination cannot be entirely ruled out. Additionally, this study did not investigate the origin or the precise pathways through which these bacteria infiltrate thrombi. The role of bacterial proteins including SdrG and ElrA in venous thrombus formation needs to be studied using molecular methods. Understanding these mechanisms could provide valuable insights into how bacterial presence affects thrombus composition and subsequent clinical outcomes.

Further studies are needed to explore the potential therapeutic implications of these findings. For instance, if certain bacteria are found to consistently influence thrombus composition and stability, targeted antimicrobial therapy could become a complementary strategy to mechanical thrombectomy in managing acute ischemic stroke.

## 5. Conclusions

This study provides evidence of a diverse bacterial community within thrombi from AIS patients, with *Corynebacterium* spp. as the most prevalent genus. The implications of these findings for stroke pathogenesis and treatment strategies warrant further investigation. Understanding the role of these bacterial communities in clot formation and stability could pave the way for novel therapeutic approaches in the management of ischemic stroke. Future research should aim to explore these mechanisms in greater detail, with larger sample sizes and a focus on the clinical implications of microbial presence in thrombi.

## Figures and Tables

**Table 1:** Characteristics of included patients in the study.

Characteristic	N = 20
**Male Gender**	10 (50%)
**Race**	
Black or African American	1 (5.0%)
Other	1 (5.0%)
White	18 (90%)
**Ethnicity**	
Central American	1 (5.0%)
Hispanic or Latino	1 (5.0%)
Not Hispanic or Latino	18 (90%)
**Age**; Median (Q1, Q3)	70 (61, 77)
**tPA Usage**	8 (40%)
**Comorbidities**	
HTN	15 (75%)
DM	2 (10%)
HLP	11 (55%)
CAD	6 (30%)
AF	10 (50%)
CHF	5 (25%)
COPD	5 (25%)
Previous Stroke	3 (15%)
**Current Smoker**	15 (75%)
**No. Passes**	
1	11 (55%)
2	5 (25%)
3	2 (10%)
6	1 (5.0%)
Multiple (More Than 5)	1 (5.0%)
**Recanalization (TICI)**	
2B	5 (25%)
3	15 (75%)
**Vessel Occluded**	
BA	1 (5.0%)
ICA	2 (10%)
ICA and M1	2 (10%)
M1	8 (40%)
M1 and M2	3 (15%)
M2	4 (20%)
**Side**	
Left	7 (37%)
Right	12 (63%)
Unknown	1
**History of Antibiotic Treatment**	1 (5.0%)

HTN: Hypertension; DM: Diabetes Mellitus; HLP: Hyperlipidemia; CAD: Coronary Artery Disease; AF: Atrial Fibrillation; CHF: Congestive Heart Failure; COPD: Chronic Obstructive Pulmonary Disease; tPA: Tissue Plasminogen Activator; TICI: Thrombolysis in Cerebral Infarction; BA: Basilar Artery; ICA: Internal Carotid Artery; M1: First Segment of Middle Cerebral Artery; M2: Second Segment of Middle Cerebral Artery.

**Table 2:** Underlying conditions, antibiotic history, and inflammatory biomarkers of the study cohort.

Characteristic	N = 20
**History of high-risk infectious diseases**	
HCV	0 (0%)
HIV	1 (0%)
Gingivitis	2 (0%)
Gut dysbiosis	3 (0%)
**History antibiotics during admission**	
Cefepime and vancomycin and cefazolin	1 (5.0%)
Levofloxacin	1 (5.0%)
Nitrofurantoin, cefdinir, piperacillin, and tazobactam	1 (5.0%)
Cefazolin	1 (5.0%)
**Immunedeficiency**	
Breast cancer	2 (10%)
Gulucocorticoid use due to lymphocytic colitis	1 (5.0%)
Malignant nodule of lung	1 (5.0%)
Confirmed bacterial endocarditis	0 (0%)
**Positive blood culture**	
Candida	1 (5.0%)
Staphylococcus epidermis	1 (5.0%)
**Biomarkers of inflammation**	
Leukocytosis	8 (40%)
Increased CRP	1 (5.0%)
increased lactate	1 (5.0%)
leukocytosis, increased lactate and CRP	1 (5.0%)
**Stroke etiology**	
Thromboembolism	16 (80%)
Athrosclerosis	3 (15%)
Athrosclerosis and distal emboli	1 (5.0%)

HCV: Hepatitis C Virus; HIV: Human Immunodeficiency Virus; CRP: C-Reactive Protein.

## Data Availability

The data that support the findings of this study are available from the corresponding author upon reasonable request due to patient privacy and ethical considerations.

## Supplementary Materials

The following supporting information can be downloaded at https://www.mdpi.com/article/10.3390/brainsci15020157/s1, Figure S1: Genome characteristics exemplifying the presence of bacterial genetic material in metagenome isolated from stroke patients’ thrombi (A) Metagenome (B) Amplicons of 16S rDNA region (C) Amplicons of housekeeping gene GAPDH; Figure S2: Fluorescence in situ hybridization (FISH) analysis; Table S1: Details on the primers used for species confirmation; Table S2: Summary of sample characteristics, including sequencing depth and group assignment; Table S3: Bacterial ASVs (A) Before filtering with 25 percentile Cutoff values (B) After filtering with 25 percentile Cutoff values; Table S4: Proportions of bacterial taxa within each sample.; Table S5: Alpha diversity indices (Shannon and Simpson) for each sample; Table S6: Beta diversity distances (Bray-Curtis dissimilarity) between samples.
