# Supplementary material for: Metagenomic Insights into Microbial Signatures in Thrombi from Acute Ischemic Stroke Patients Undergoing Endovascular Treatment
Source: Brain Sci. 2025 Feb 6;15(2):157. doi: 10.3390/brainsci15020157 (PMC11853128; doi:10.3390/brainsci15020157)
Supplement: Supplementary file 1 [file brainsci-15-00157-s001.zip › Figure S1.pdf]

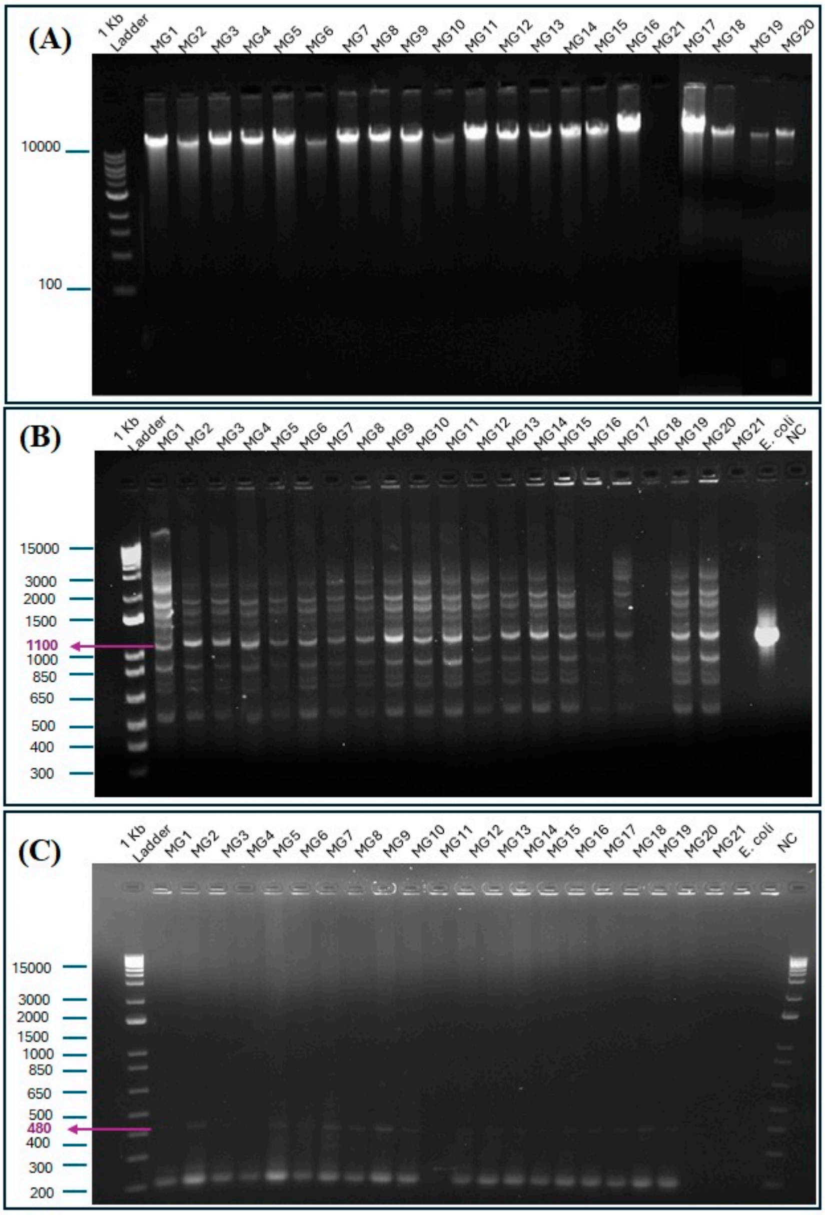

MG21 – Sample negative control (PBS), NC – Reaction negative control

Figure S1. Genome characteristics exemplifying the presence of bacterial genetic material in metagenome isolated from stroke patients' thrombi (A) Metagenome (B) Amplicons of 16S rDNA region (C) Amplicons of housekeeping gene GAPDH.
