# Supplementary figures and images for: Metagenomic Insights into Microbial Signatures in Thrombi from Acute Ischemic Stroke Patients Undergoing Endovascular Treatment
Source: Brain Sci. 2025 Feb 6;15(2):157. doi: 10.3390/brainsci15020157 (PMC11853128; doi:10.3390/brainsci15020157)

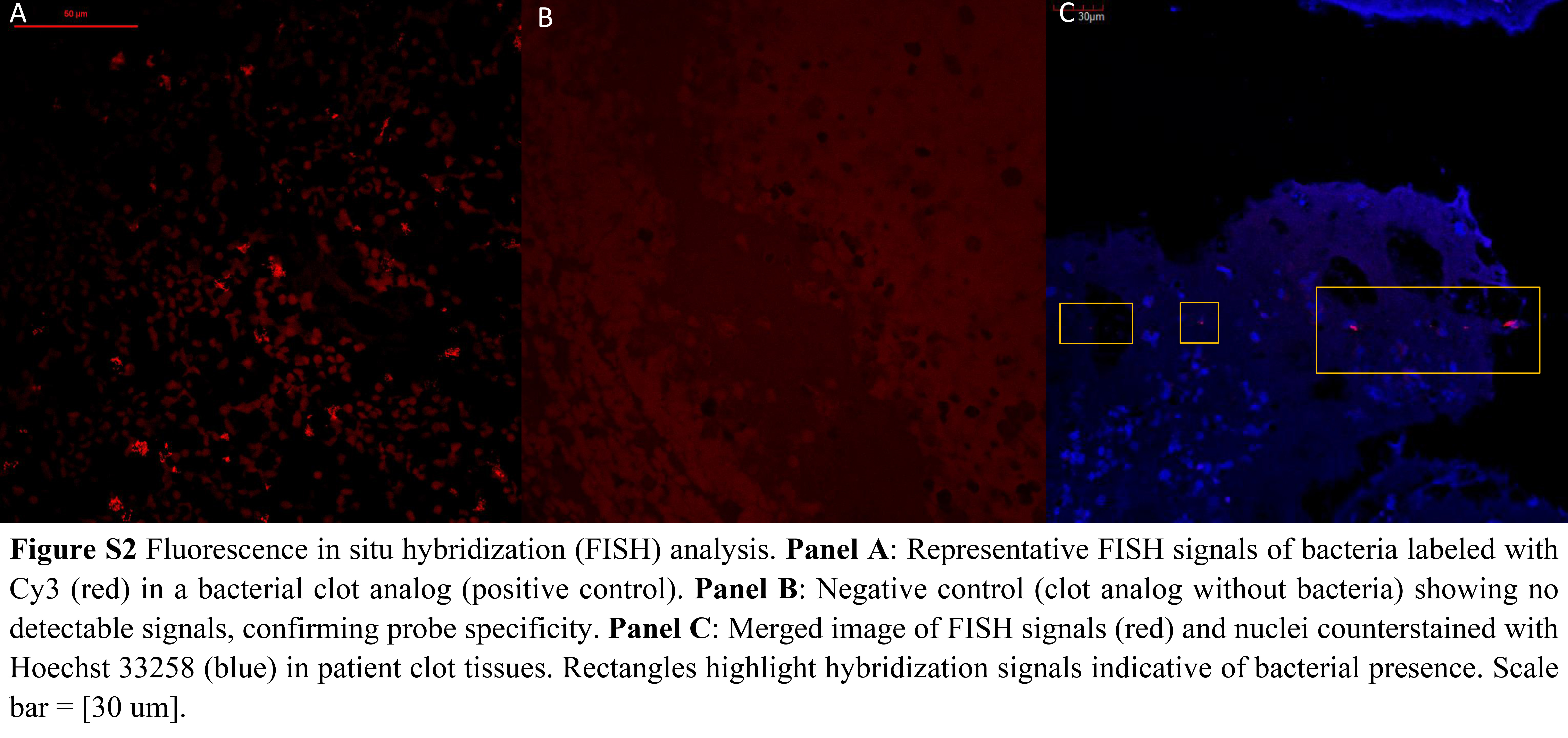

Supplement: Supplementary file 1 [file brainsci-15-00157-s001.zip › Figure S2.jpg]
